# Lupus Nephropathy in Mauritania: A Clinical and Longitudinal Study from the National Hospital Center of Nouakchott

**DOI:** 10.7759/cureus.90636

**Published:** 2025-08-21

**Authors:** Mohamed Lemrabott, Sidi Mohamed Mah, Jemal Awa, Mohamed Yahya Sidi Hamoud, Mohameden Meyine, Abdallahi Abed, Aminetou El Atigh, Behah Ehbibi, Yahya Izidbih, Abdellatif Sidi Aly

**Affiliations:** 1 Nephrology Department, National Hospital Center of Nouakchott, Nouakchott, MRT; 2 Nephrology Department, Faculty of Medicine, Pharmacy, and Odonto-Stomatology, University of Nouakchott, Nouakchott, MRT; 3 Nephrology Department, Hospital of Amity, Nouakchott, MRT; 4 Radiology Department, Hospital Center of Specialties, Nouakchott, MRT

**Keywords:** epidemiology, lupus nephritis, mauritania, renal pathology, systemic lupus erythematosus

## Abstract

Background and aim: Lupus nephritis (LN) is a severe complication of systemic lupus erythematosus (SLE) with variable outcomes. This study aimed to describe the epidemiological, clinical, paraclinical, histopathological, and treatment outcomes of LN in the Nephrology Department of the National Hospital Center of Nouakchott.

Methods: We conducted a cross-sectional study over a 12-month period, from March 2023 to March 2024. All included patients had systemic lupus erythematosus complicated by nephropathy. We studied the epidemiological, clinical, paraclinical, histopathological, and evolutionary profile under treatment.

Results: Twenty-five patients were included; the mean age was 35.6 years, and the sex ratio (male/female) was 0.13. Extrarenal signs were dermatological in 12 patients and rheumatological in 10 patients. The mean proteinuria was 4.11±3.18 g/24 h (range: 0.38-14.5 g/24 h). The mean serum creatinine was 46.4±53.2 mg/L (range: 5-229 mg/L), and it was elevated in 16 patients (64%). Anti-dsDNA antibodies were positive in 16 patients, and antinuclear antibodies were positive in 13 patients.

Histopathologically, classes IV and III were most frequently found in 11 and six patients, respectively; four patients had combined class IV and V, and five patients had isolated vascular and/or tubulointerstitial involvement. During the induction phase of treatment, 20 patients were on mycophenolate mofetil, while only one patient was on cyclophosphamide. Of the 22 patients with regular follow-up, three achieved complete remission, five had partial remission, nine progressed to end-stage renal disease, and five patients died.

Conclusions: Lupus nephritis often presents as severe proliferative forms, with an unpredictable course and a risk of progression to chronic kidney disease and death.

## Introduction

Lupus nephritis (LN) represents a severe complication of systemic lupus erythematosus (SLE), characterized by inflammation of kidney tissues leading to glomerular, vascular, and tubulointerstitial involvement [1]. Systemic lupus erythematosus is a multisystem disease that can affect multiple organs, as outlined in recently revised classification criteria [2]. Indications for renal biopsy remain broad in systemic lupus erythematosus due to the frequent discordance between histological severity and laboratory findings.

Renal involvement in systemic lupus erythematosus is more common in Asian (55%), African (51%), and Hispanic (43%) populations compared to Caucasians (14%) [3]. In South Africa, lupus nephritis is found in 43.8% of patients with systemic lupus erythematosus and constitutes the main risk factor for death [4]. In some African countries, the frequency of lupus nephritis is estimated between 69 and 72%, with a mortality rate ranging from 7.8 to 9.3% [5,6]. Renal involvement in lupus is a major determinant of prognosis and is associated with increased morbidity and mortality [7].

Glomerular lesions in lupus are polymorphic and complex. The International Society of Nephrology/Renal Pathology Society (ISN/RPS) 2003 histological classification, published approximately 20 years ago and updated in 2018, is used for categorizing lupus glomerulonephritis into six major classes, with an update to the classification in 2018 [8,9]. It reproducibly classifies lupus glomerulonephritis into one of the six major categories. Beyond glomerular classification, identifying associated vascular and tubulointerstitial lesions remains essential, as these can influence prognosis and guide therapeutic choices [10].

Therapeutic advances have significantly improved the five-year renal survival in patients with lupus nephritis, from 44% in the 1960s-1970s to 82% in the 2000s, and nearly 96% at present [11]. Unfortunately, lupus nephritis predominantly affects women of childbearing age, and current treatments are associated with multiple side effects. This study aimed to describe the epidemiological, clinical, paraclinical, histopathological, therapeutic, and outcome profiles of lupus nephritis in the Nephrology Department of the National Hospital Center of Nouakchott, Mauritania.

## Materials and methods

Study design, population, and sample size

This was a cross-sectional study conducted over 12 months from March 2023 to March 2024 in the Nephrology Department of the National Hospital Center of Nouakchott, a public referral health institution in Mauritania. All medical records of patients aged 14 years and older with systemic lupus erythematosus and confirmed nephropathy were included in the study. The diagnosis of SLE was based on the 2019 European Alliance of Associations for Rheumatology/American College of Rheumatology (EULAR/ACR) classification criteria [2]. The diagnosis of LN was established in cases of a proteinuria/creatinine ratio (PCR) of >0.5 g/g, proteinuria >0.5 g/24 h, active urinary sediment, or decreased glomerular filtration rate without any cause other than lupus, warranting renal biopsy [12]. Patients with incomplete medical records were excluded from this analysis.

Study measures

For each selected patient, we collected comprehensive data including epidemiological, clinical, biological, histological, therapeutic, and outcome parameters. The glomerular filtration rate (GFR) was estimated using the modified Modification of Diet in Renal Disease (MDRD) formula. Kidney biopsies were evaluated by a qualified renal pathologist and classified according to the ISN/RPS classification system.

Complete and partial remission, relapse, and resistance were defined according to the EULAR/European Renal Association-European Dialysis and Transplant Association (ERA-EDTA) criteria [13]. Complete remission was defined as daily proteinuria <0.5 g or PCR <50 mg/mmol, with a normal GFR or a GFR no more than 10% below normal values. Partial remission was defined as a reduction in proteinuria of more than 50%, daily proteinuria <3 g, and a normal or near-normal GFR, preferably within six months but not later than 12 months after induction therapy.

Treatment resistance was defined as either a lack of clinical improvement within three to four months of therapy initiation or the absence of partial remission after six to 12 months of treatment. Disease relapse was characterized by an increase in serum creatinine ≥30% or a decrease in GFR ≥10% with active urinary sediment, or alternatively by PCR >100 mg/mmol (proteinuria >1 g) after complete remission, or >200 mg/mmol (proteinuria >2 g) after partial remission.

Statistical analysis

Data were collected using standardized survey sheets from patient medical records. Data entry and statistical analysis were performed using Microsoft Excel 2024 (Redmond, WA: Microsoft Corp.) and Statistical Package for the Social Sciences (SPSS) version 18 software (Armonk, NY: IBM Corp.).

## Results

Patient demographics and baseline characteristics

During the study period, 25 cases of lupus nephritis were identified, and only 22/25 patients (88%) had regular follow-up and were included in the final analysis. The mean age of the patients was 35.6±12.3 years (range: 14-60 years). The 21-30 years age group was the most represented, accounting for 9/25 cases (36%). There were 22/25 women (88%) and 3/25 men (12%), with a sex ratio of 0.13. Three patients (12%) came from semi-urban areas, and 22/25 patients (88%) from urban areas.

Clinical presentation

Systemic lupus erythematosus was previously known in 4/25 patients (16%), while lupus nephritis was the initial presentation in 21/25 patients (84%). Extrarenal manifestations were predominantly dermatological in 12/25 patients (48%) and rheumatological in 10/25 patients (40%). Two patients (8%) presented with ascites, and another 2/25 patients (8%) had pleuritis (Table 1).

**Table 1:** Epidemiological and clinical characteristics of patients. SLE: systemic lupus erythematosus; M: male; F: female

Variables	Results
Total number of identified cases	25
Number with regular follow-up	22
Mean age±standard deviation	35.6±12.29 years
Most represented age group (21-30 years)	36%
Female sex	22 (88%)
Male sex	3 (12%)
Sex ratio (M/F)	0.13
Urban origin	88%
Semi-urban origin	12%
Lupus nephropathy as initial presentation of SLE	21 (84%)
Previous diagnosis of SLE before nephropathy	4 (16%)
Extra-renal dermatological manifestations	12 (48%)
Extra-renal rheumatological manifestations	10 (40%)
Ascites	2 (8%)
Pleural effusion	2 (8%)

Laboratory findings

**Figure 1:**
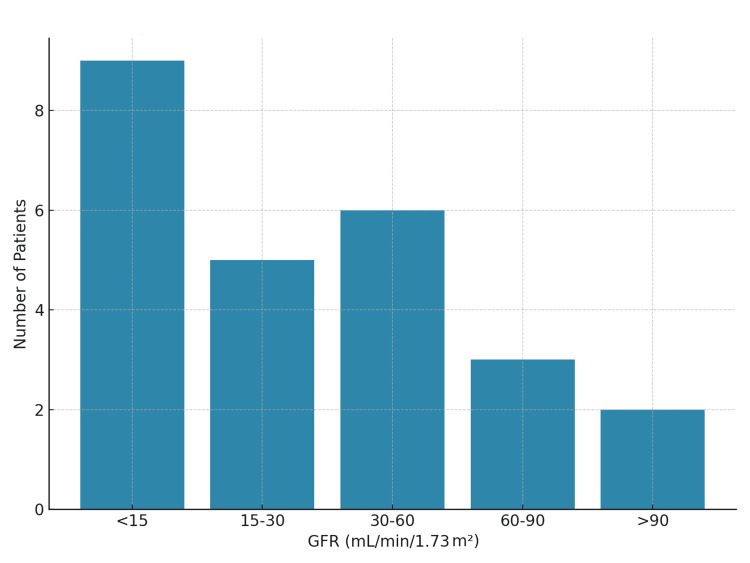
Distribution of GFR values among patients. GFR: glomerular filtration rate

Proteinuria values ranged from 0.38 g to 14.5 g/24 h, with a mean of 4.11±3.18 g/24 h. Twelve patients (54%) exhibited proteinuria levels exceeding 3 g/24 h, consistent with the nephrotic range. The mean serum creatinine was 46.4±53.2 mg/L (range: 5-229 mg/L), and it was elevated in 16/25 patients (64%). The mean glomerular filtration rate (GFR) was 48.7±47.7 mL/min/1.73 m² (range: 4.3-139 mL/min/1.73 m²). GFR was <15 mL/min/1.73 m² in 9/25 patients (36%) (Figure 1).

Immunological profile

Anti-dsDNA antibodies were positive in 16/23 patients (69.6%) among those tested. Anti-nucleosome and anti-Sm antibodies were each positive in 1/25 patients (4%), anti-SSa antibodies in 1/25 patients (4%), and anti-SSb antibodies in 2/25 patients (8%). Complement levels were measured in 19/25 patients (76%); C3 was decreased in 10/19 patients (52.6%), and C4 in 8/19 patients (42.1%) (Table 2).

**Table 2:** Biological data of patients.

Variables	Results
Mean proteinuria (g/24 h)	4.11±3.18 (0.38-14.5)
Mean serum creatinine (mg/L)	46.4±53.15 (5-229)
Elevated serum creatinine	16 patients (64%)
Positive anti-nucleosome Ab	1 patient
Positive anti-Sm Ab	1 patient
Positive anti-SSa Ab	1 patient
Positive anti-SSb Ab	2 patients
Complement measured	19 patients
Decreased C3	10 patients
Decreased C4	8 patients

Histological findings

**Figure 2:**
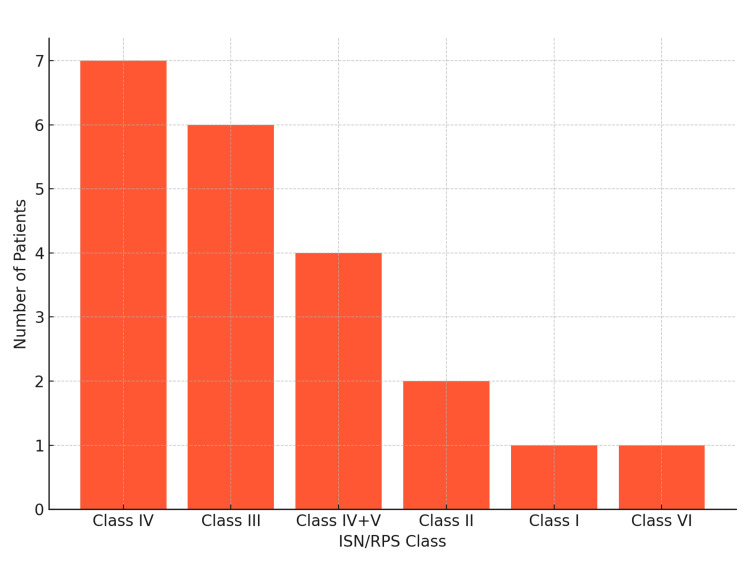
Distribution by ISN/RPS classification. ISN/RPS: International Society of Nephrology/Renal Pathology Society

Twenty-two patients (88%) underwent renal biopsy for various indications (Table 3). Class IV was the most frequent histological pattern, found in 7/22 patients (31.8%). Four patients (18.2%) had combined class IV and V, and 6/22 patients (27.3%) had class III. Class II was identified in 2/22 patients (9.1%), while classes I and VI were each found in 1/22 patient (4.5%) (Figure 2).

**Table 3:** Indications of renal biopsies.

Indications	Number	Percentage
Pure nephrotic syndrome	12	54.0
Impure nephrotic syndrome	6	27.22
Impaired renal function	1	4.54
Known lupus with proteinuria	3	13.6

Five patients (22.7%) had isolated vascular and/or tubulointerstitial involvement. Immunofluorescence was performed in 18/22 patients (81.8%), revealing IgM, IgG, and C3 deposits in 12/18 patients (66.7%), IgA in 1/18 patient (5.6%), and C1q in 7/18 patients (38.9%).

Treatment regimens

During the induction phase, 20/25 patients (80%) received methylprednisolone pulses over three days followed by oral prednisone. Immunosuppressants were administered to 21/25 patients (84%), of whom 20/21 patients (95.2%) received mycophenolate mofetil and 1/21 patient (4.8%) received cyclophosphamide. For maintenance therapy, 17/25 patients (68%) continued corticosteroids, and immunosuppressants were maintained in 22/25 patients (88%), with 21/22 patients (95.5%) on mycophenolate mofetil and 1/22 patient (4.5%) on azathioprine.

Clinical outcomes

Over the six-month follow-up period, there were 2/25 cases (8%) of complete remission, 2/25 cases (8%) of partial remission, 2/25 relapses (8%), and 2/25 deaths (8%). Nine patients (36%) required dialysis, and no patient received a kidney transplant during this period. During the 12-month follow-up, 9/25 patients (36%) progressed to end-stage renal disease, of whom 5/9 patients (55.6%) died and 4/9 patients (44.4%) remained on hemodialysis (Table 4).

**Table 4:** Clinical evolution of patients at six months and 12 months. CKD: chronic kidney disease

Evolution	Month 6	Month 12	Total
Complete remission	2	1	3
Partial remission	2	3	5
Relapse	2	3	5
CKD	4	5	9
Death	3	2	5

## Discussion

Epidemiological characteristics

Our series found an average age of 35.6±12.27 years, comparable to that found by Ka et al. and Mansour et al. [5,6]. This average age falls within the typical age range for the onset of lupus nephropathy, mainly between 15 and 45 years [1]. The sex ratio of 0.13 observed in our study confirms the characteristic female predominance of this pathology, with a ratio of nine women to one man, consistent with international literature data [1]. A particularly notable aspect of our series is that nephropathy was the presenting manifestation of lupus disease in 21/25 patients (84%), a result comparable to that of Ebana et al., who reported 90% of concomitant diagnosis [14]. This high proportion probably suggests a diagnostic delay or insufficient awareness of the early manifestations of systemic lupus erythematosus in our setting.

Clinical manifestations

The extra-renal manifestations were predominantly dermatological in 12/25 patients (48%) and articular involvement in 10/25 patients (40%). These results, although slightly lower than those found by Ka et al. (67% and 60% respectively), remain consistent with the classic presentation profile of lupus disease [5].

Biological and histopathological data

The mean serum creatinine level of 46.4 mg/L in our series was higher than that reported by Ka et al. (21 mg/L) and Ebana et al. (17 mg/L) [5,14]. This difference could be explained by a later diagnosis in our context, with 16/25 patients (64%) having impaired renal function at the time of diagnosis, a proportion higher than that of Ka et al. (40%) but comparable to that of Béji et al. (51%) [5,15]. The average proteinuria of 4.11±3.18 g/24 h is higher than that reported by Ka et al. and Béji et al. and comparable to that of Gassongo-Koumou et al. [5,15,16]. This significant proteinuria reflects the severity of glomerular damage at the time of diagnosis.

The histopathological study, performed in 22/25 patients (88%), showed a predominance of proliferative classes, with 7/22 patients (31.8%) having class IV and 6/22 patients (27.3%) having class III. These results are comparable to those reported by Béji et al. for class III and similar to those by Niang et al. for class IV [15,17]. The association of classes IV+V was present in 4/22 patients (18.2%). Notably, isolated class V was absent in our series, contrary to what is generally reported in the literature, which could reflect a selection of more severe cases in our cohort. Class VI was present in 1/22 patients (4.5%), which is comparable to that found by Béji et al. and Okpechi et al. [15,18]. The high frequency of proliferative forms (classes III and IV) in our series could reflect the frequent delays in diagnosis and management of systemic lupus in our setting, leading to silent progression of renal lesions towards severe active forms. Moreover, the delayed recourse to renal biopsy, often limited by technical and socioeconomic constraints, may contribute to identifying patients only at the stage of advanced proliferative nephropathy.

Therapeutic aspects

Immunosuppressive treatment was dominated by mycophenolate mofetil in 20/21 patients (95.2%) who received immunosuppressants, contrasting with the studies by Mansour et al. and Béji et al., which reported 38.8% and 1%, respectively [6,15]. The predominance of mycophenolate mofetil use in our cohort reflects its greater availability, concerns regarding severe infections previously observed with cyclophosphamide in vasculitis cases, and established physician practice patterns in our center. Although both drugs remain recommended by international guidelines for proliferative lupus nephritis, local clinical experience and practice habits often guide the therapeutic choice. All patients included in the study received corticosteroid therapy, in accordance with international recommendations [19,20]. Maintenance treatment combined low-dose corticosteroids and immunosuppressants in all patients followed.

Evolution and prognosis

The evolution of our patients reveals a concerning prognosis, with 9/25 patients (36%) progressing to end-stage chronic renal failure and 5/25 deaths (20%) over 12 months of follow-up. These results contrast unfavorably with data from developed countries, where the risk of end-stage renal failure at five years is 11% and overall survival at 10 years ranges between 92% and 98% [20]. In the United States, ethnic differences have been documented with lower 10-year renal (68% vs. 38%) and overall survival rates (81% vs. 59%) for black patients compared to Caucasian patients [21,22]. Unfortunately, our results align with this trend observed in populations of African origin. Several factors can explain these unfavorable outcomes as follows: late diagnosis with already advanced histological lesions, the presence of fibrosis in 7/22 patients (31.8%) among those biopsied (higher than the 18% reported by Béji et al.), and possibly socioeconomic factors limiting access to care and therapeutic adherence [15].

Study limitations

Our study has several limitations that should be mentioned. From a methodological perspective, this was a monocentric study, which limited the representativeness of the results at the national level due to its relatively small sample size of 25 patients, thereby reducing statistical power. The cross-sectional design did not allow the establishment of causal relationships, and the follow-up duration was limited to 12 months, which was insufficient to assess long-term prognosis.

Technical and contextual limitations included loss to follow-up of 3/25 patients (12%), which could introduce selection bias, and limited access to certain specialized complementary examinations, with immunofluorescence performed in only 18/22 patients (81.8%) who underwent biopsy. There was an absence of systematic measurement of certain specific autoantibodies, and economic and geographical constraints were potentially responsible for late diagnoses. The absence of a renal histopathologist in the country, with interpretation of samples abroad, resulted in the absence of a clear reference standard for interpretation. Missing data occurred for certain biological parameters, with complement measured in only 19/25 patients (76%).

Despite these limitations, this Mauritanian study on lupus nephropathy provides valuable data for understanding this pathology in sub-Saharan Africa. It emphasizes the need for earlier diagnosis, strengthening of diagnostic and therapeutic capacities, as well as improved access to specialized care. Multicenter studies with larger sample sizes and prolonged follow-up would be necessary to confirm these results and better define therapeutic strategies adapted to the African context. The establishment of national or regional registries would also allow for better epidemiological surveillance of this serious disease.

## Conclusions

This study, conducted in Mauritania, describes the profile of lupus nephropathy, revealing frequently severe renal damage. Histological classes IV and III predominated, associated with high proteinuria and frequent impairment of renal function. Mycophenolate mofetil was the predominant treatment, with variable outcomes ranging from remission to end-stage renal disease. Mortality and progression to end-stage chronic kidney disease highlight the often poor prognosis of this complication. At the policy level, we recommend national awareness campaigns, targeted training for primary care physicians, and the establishment of specialized lupus clinics to improve early detection, timely referral, and standardized management of lupus nephritis in Mauritania. Additional studies are necessary to refine local therapeutic strategies. Finally, these data contribute to improved epidemiological knowledge of lupus nephritis in sub-Saharan Africa.
